# Beyond implementation: Uncovering the parallels between de-implementation and antimicrobial stewardship

**DOI:** 10.1017/ash.2023.150

**Published:** 2023-04-17

**Authors:** Sonali D. Advani, Virginia McKay

**Affiliations:** 1 Department of Medicine, Division of Infectious Diseases, Duke University School of Medicine, Durham, North Carolina; 2 Brown School, Washington University in St. Louis, St. Louis, Missouri

## Abstract

De-implementation is defined as the process of discontinuing, removing, reducing, or replacing a harmful, ineffective, or low-value clinical practice or intervention. The goal of de-implementation strategies is to minimize patient harm, maximize use of resources, and reduce healthcare costs and inequities. Both antibiotic and diagnostic stewardship programs focus on reducing low-value interventions (tests or antimicrobials). Stewardship interventions commonly involve de-implementation and deprescribing strategies. This commentary explores unique aspects of deimplementing low-value testing and unnecessary antimicrobial use, similarities between de-implementation and stewardship approaches, multilevel factors that impact de-implementation, and opportunities for future research.

Approaches that promote high-value care and reduce low-value practices have gained more visibility in recent times. De-implementation is defined as the process of discontinuing, removing, reducing or replacing a low-value clinical practice or intervention.^1^ Low-value practice is an umbrella term used to define practices that may be harmful, ineffective, wasteful, or neither beneficial to the patient nor cost effective.^2,3^ Similarly, deprescribing is the process of reducing or stopping medications that pose more harm than benefit or are no longer necessary.^4,5^ One target for deimplementing low-value testing and deprescribing antibiotics is asymptomatic bacteriuria. The goal of de-implementation and deprescribing strategies is to minimize patient harm, to maximize use of resources, and to reduce healthcare spending and inequities. De-implementation research is situated primarily within the broader field of implementation science which focuses on evidence-based strategies to promote the dissemination and implementation of evidence-based practices.

Overall, both antibiotic and diagnostic stewardship share many similarities with de-implementation and deprescribing in terms of their evidence-based strategies, goals to improve patient outcomes, the need for collaboration and multidisciplinary approach, education and training, monitoring, and sustainability. In this commentary, we explore unique aspects of deimplementing low-value practices, similarities between de-implementation and stewardship approaches, multilevel factors that impact de-implementation, and opportunities for future research.^6^ Specific examples of de-implementation approaches in antibiotic and diagnostic stewardship are described in Table 1.

**Table 1.** De-implementation Approaches in Antibiotic and Diagnostic Stewardship

De-implementation Approaches	Antibiotic Stewardship Examples^22^	Diagnostic Stewardship Examples^18^
Reduction (in frequency and/or intensity)	Shorten the duration of antimicrobial therapy	Reduction in urine culture orders using an evidence-based algorithm
Restriction	Formulary restriction	Suppression of low-yield urine culture results in electronic medical records (require clinicians to call the laboratory if result was desired)
Discontinuation without replacement	Stop antibiotic completely	Hard stop for test (urine culture order cancelled if pyuria is absent on preceding urinalysis)
Discontinuation with replacement	Parenteral to oral antibiotic conversion	Performing “urinalysis with reflex to culture” instead of “direct urine culture”

## Steps in de-implementation

There is little guidance on de-implementation of ineffective, low-value, or harmful healthcare practices.^7^ Niven et al^2^ proposed a framework for conceptualizing de-implementation which typically include the following steps:

*Phase 1: Identify the low-value practice.* This step involves identifying the low-value practice that should be discontinued, reduced or replaced.^2^ (eg, identifying the overuse of urine cultures in asymptomatic patients)

*Phase 2: Assessing barriers and facilitators.* Once the low-value practice has been identified, the next step is to assess barriers and facilitators to discontinuing the practice, preferably using a framework. For example, the Theoretical Domains Framework (TDF) or Capability Opportunity Motivation model of Behavior (COM-B model)^8,9^ can be used to assess barriers and facilitators to stop unnecessary urine-culturing by evaluating capability (ie, limited knowledge and skills), opportunity (ie, preselected orders, time and pharmacist support), and motivation (ie, belief about risk and nurse and patient expectations).^9,10^

*Phase 3: Developing a de-implementation strategy.* The intervention should target specific barriers identified using prior models or frameworks (eg, COM-B model or TDF framework). Additionally, it is important to assess whether this low-value practice can be completely discontinued, reduced, restricted, or replaced with a different practice. Based on the COM-B model, if preselected urine culture orders in admission order sets was identified as the primary driver of inappropriate prescribing, then environmental restructuring (removing preselected orders) could serve as the primary de-implementation approach.

**Fig. 1.**
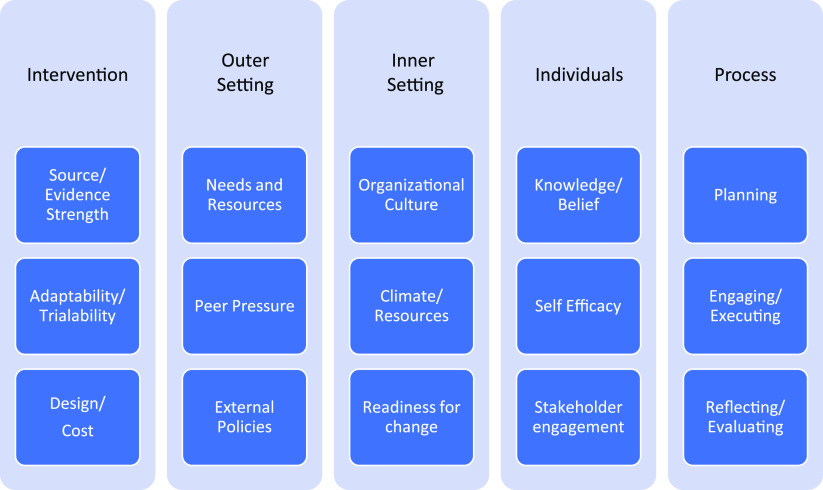
Consolidated Framework for Intervention Research (CFIR) constructs for implementation and de-implementation.

*Phase 4: Strategy adoption.* This step involves organizational assessment,^11^ stakeholder engagement, educating and training healthcare staff, and making any necessary policy or procedural changes.^3^ At this stage, we recommend using one of the implementation science theories, models or frameworks, e.g. the Consolidated Framework for Implementation Research (CFIR) to assess multilevel factors and produce actionable results using an iterative process (Fig. 1).^12^

*Phase 5: Strategy evaluation.* Evaluation is an iterative process involves monitoring the de-implementation process, assessing its impact by analyzing outcomes (quantitative and or qualitative), and making any necessary alterations to the plan. At this stage, a framework like Reach, Effectiveness, Adoption, Implementation, and Maintenance (RE-AIM)^13^ can be used to perform a comprehensive evaluation. For example, RE-AIM can be used to measure the change in urine culture utilization and antimicrobial use after removal of preselected urine cultures from an order set to assess the effectiveness of the de-implementation strategy.

*Phase 6: Scaling and sustainability.* This step involves scaling to other sites if applicable (with continued evaluation), and long-term discontinuation of the low-value practice by regular monitoring, reinforcement, assessing work arounds and education.

## De-implementation theories

Several theories, models, and frameworks, including those drawn from other disciplines like psychology (classic theories) and those developed or adapted for use in implementation science (implementation theories), can be applied to de-implementation of low-value practices.^14^ Although several theories and models are used to guide de-implementation, we describe a few pertinent theories below that can be applied to stewardship.

*Theory of planned behavior (TBP).* This theory proposes that behavior is influenced by 3 core components: attitude, subjective norms, and perceived behavioral control. TPB is one of the most commonly used theories, and 25%–34% of the variance in behavior can be explained by this theory.^10^

*Theoretical framework to understand appropriateness of primary care provider service recommendations.* This theory by Powell et al^15^ is informed by TBP, and includes 5 determinants: clinician beliefs, assessment intentions, assessment of the appropriateness of the intervention, appropriate recommendations, and patient acceptance of the recommendations.

*Framework for understanding and reducing medical overuse.* This theory by Morgan et al^16^ is informed by factors contributing to variations in physicians’ use of evidence at the point of care, and includes 6 determinants: culture of healthcare consumption, culture of professional medicine, practice environment, patient factors and experience, clinician attitudes and beliefs, and patient clinician interaction.

*Diffusion of innovations theory.* The diffusion of innovation (DOI) theory is the spontaneous process by which new innovations are first adopted by innovators and early adopters (ie, ‘leaders’), followed by an early majority, a late majority, and laggards. However, leaders in de-implementation may have been sceptics of the practice in question.^17^ Additionally, at least some early adopters in implementation might also be early deimplementers, as they tend to be open to new evidence, to have a high degree of opinion leadership, and to generally be respected by their peers.^17^

*The normalization process theory (NPT).* This theory helps explain how new practices are embedded in the daily routine of clinicians and organizations. Based on this theory, de-implementation efforts should focus on understanding how the practice or intervention is embedded in the daily routine and creating a process to un-embed it.^10^

These theories discussed above promote understanding of underlying factors that influence clinician and organizational behavior, and they can be used to design effective stewardship interventions directed toward discontinuing unnecessary testing and antibiotics. For example, understanding that increase in urine tests is driven by inclusion of screening urinalysis orders (NPT theory) in admission or presurgical order sets allows de-implementation efforts to focus on removing these tests from admission or presurgical order sets.^18^

## Multilevel factors affecting de-implementation

Various frameworks and models have been used to describe factors that influence the pace and extent of the implementation of an innovation or intervention (eg, TDF and CFIR).^19^ Although CFIR is primarily intended to identify potential factors that influence implementation, similar factors influence the pace and extent of de-implementation. These include strength of the underlying evidence, the complexity of the intervention, external pressure, organizational culture, and fear and anxiety of individuals (patients, nurses, and clinicians) involved in changing an established clinical practice, and ultimately the process of de-implementation from planning to evaluation (Fig. 1). By following a framework like CFIR, stewardship programs can be tailored to the specific needs of the local healthcare setting. Furthermore, CFIR gives stewardship programs the ability to monitor and evaluate the progress of their intervention and allows for adjustments to be made as needed to improve outcomes.

## Future directions

The emerging field of de-implementation science holds promise for supporting antibiotic and diagnostic stewardship. Despite the availability of several frameworks and models to guide the study of implementation, more evidence is needed to gauge applicability to de-implementation due to the differences in processes involved and necessary behavior change strategies.^17,20,21^ Areas of future research for de-implementation in stewardship include better understanding of barriers and facilitators to deprescribing antibiotics, including physician, nurse, and patient factors, as well as healthcare system-level factors. More qualitative research is needed to understand how to best communicate with staff as well as patients and their families to drive change. Most importantly, we need a better understanding of the cultural and ethical implications of our stewardship interventions, including how some of our stewardship interventions may affect patient and staff autonomy as well as health disparities. Early examination of these factors will allow for long-term success and sustainability of stewardship interventions.
